# Nanostructured superhydrophobic films synthesized by electrodeposition of fluorinated polyindoles

**DOI:** 10.3762/bjnano.6.212

**Published:** 2015-10-28

**Authors:** Gabriela Ramos Chagas, Thierry Darmanin, Frédéric Guittard

**Affiliations:** 1 Univ. Nice Sophia Antipolis, CNRS, LPMC, UMR 7336, 06100 Nice, France; Fax: (+33)492076156; Tel: (+33)492076159

**Keywords:** bioinspiration, conducting polymers, electrochemistry, nanostructures, polyindoles, superhydrophobic

## Abstract

Materials with bioinspired superhydrophobic properties are highly desirable for many potential applications. Here, nine novel monomers derived from indole are synthesized to obtain these properties by electropolymerization. These monomers differ by the length (C_4_F_9_, C_6_F_13_ and C_8_F_17_) and the position (4-, 5- and 6-position of indole) of the perfluorinated substituent. Polymeric films were obtained with C_4_F_9_ and C_6_F_13_ chains and differences in the surface morphology depend especially on the substituent position. The polyindoles exhibited hydrophobic and superhydrophobic properties even with a very low roughness. The best results are obtained with **PIndole-6-F_6_** for which superhydrophobic and highly oleophobic properties are obtained due to the presence of spherical nanoparticles and low surface energy compounds.

## Introduction

The number of studies about materials with superhydrophobic properties, characterized by extremely high water contact angles (θ_w_) and low water adhesion or hysteresis (also known as “Lotus effect”), grows exponentially because of the importance for both the scientific and industrial community [1–6]. Superhydrophobic properties are quite common in nature, in both animals and plants, and allow them surviving against predators or hostile environments such as extremely humid or dry regions, for example [7–12]. Bioinspiration has shown the importance of developing structured surfaces in the presence of low surface energy materials that allow one to obtain more easily superhydrophobic properties with higher robustness [13–15]. Controlling the surface energy and the roughness is hence fundamental to achieve the superhydrophobicity.

All kind of materials can be used to reach superhydrophobicity, but conducting polymers have many advantages such as an easiness to functionalize and opto-electronic properties [16] with the possibility to introduce various dopants (smart materials) [17–18]. Conducting polymers are also exceptional materials for the control of surface nanostructures and wettability. First of all, nanostructures of extremely various shapes can be produced in solution by self-assembly [19–21] or directly formed on substrates by different strategies such as preferential growth [22], grafting [23], vapor phase polymerization [24], plasma polymerization [25] and electropolymerization [26–30]. The last method allows for a very quick and easy deposition of conducting polymer films while the formation of surface structures can be controlled by electrochemical parameters [26] and the used monomer [27]. In order to control the formation of surface nanostructures, the core responsible for the polymerization (such as thiophene, pyrrole or 3,4-ethylenedioxythiophene) [27–30] is probably the most important parameter. Then, the polymer can also be controlled by introducing hydrophobic/hydrophilic substituents or dopant agents [17–18,27–30]. In most of the cases, fluorocarbon or hydrocarbon chains were used to reach superhydrophobic properties.

**Scheme 1:**
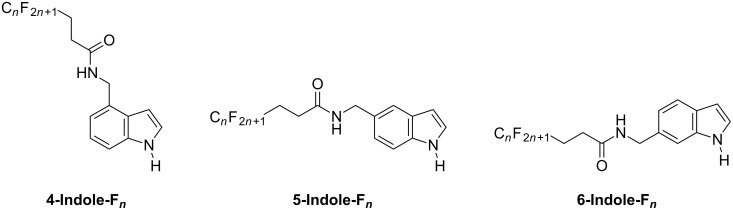
Original indole monomers synthesized and used in this manuscript.

Here, we report for the first time the formation of superhydrophobic properties from indole derivatives. Nine novel indole monomers substituted by fluorocarbon chains of different length (C_4_F_9_, C_6_F_13_ and C_8_F_17_) and in different positions (4-, 5- and 6-position of indole) were synthesized and electropolymerized (Scheme 1). We report the influence of the fluorocarbon chain length and the substituent position on the surface morphology and hydrophobicity.

## Results and Discussion

### Electrodeposition

**Figure 1:**
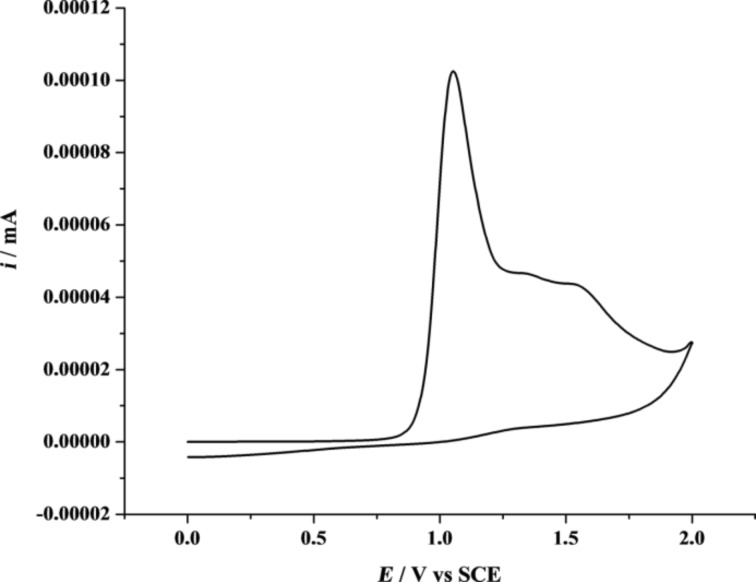
Cyclic voltammogram of the monomer **Indole-6-F_6_** (1 scan at 20 mV·s^−1^). Electropolymerization in 0.1 M of acetonitrile/Bu_4_NClO_4_.

**Figure 2:**
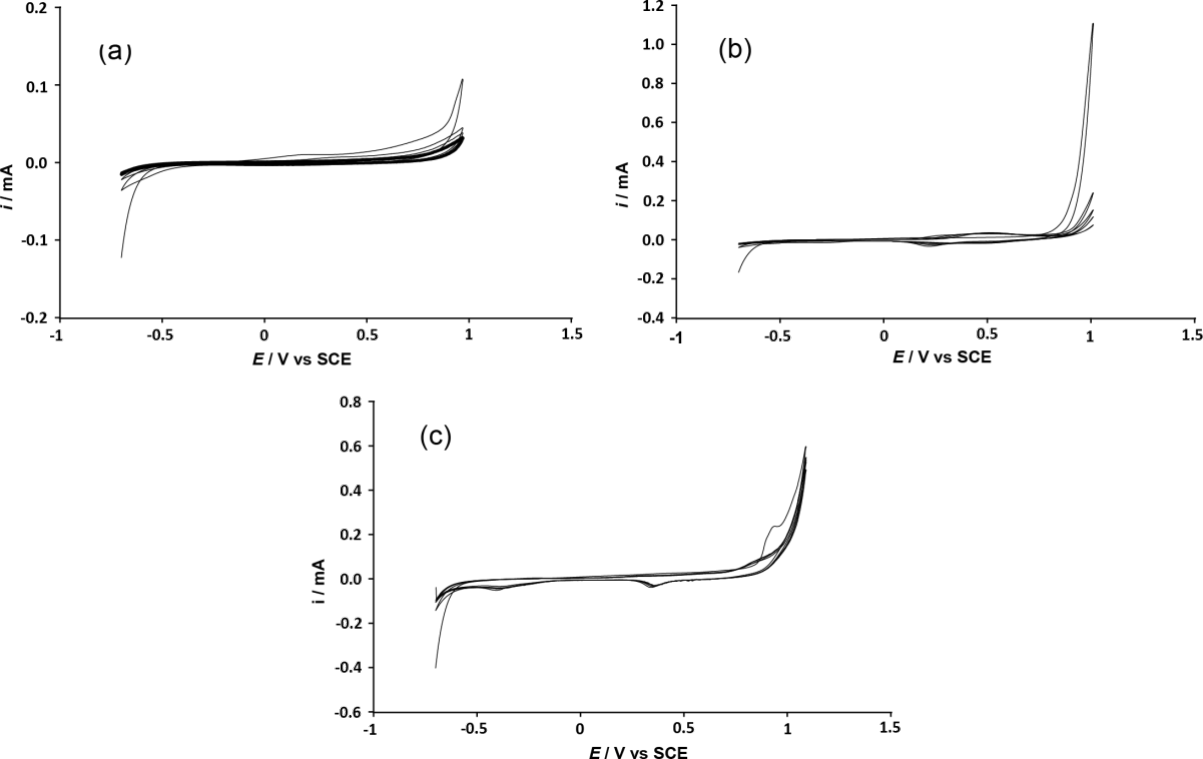
Cyclic voltammogram of polyindoles. (a) **Indole-6-F_8_**, (b) **Indole-6-F_6_** and (c) **Indole-6-F_4_** (5 scans at 20 mV·s^−1^). Electropolymerization in 0.1 M of acetonitrile/Bu_4_NClO_4_.

In order to develop structured polymeric films, the monomers were electropolymerized. First of all, it was necessary to determine the oxidation potential (*E*^ox^) of all the monomers. These *E*^ox^ were determined by cyclic voltammetry and were found to be in the range of 0.9–1.3 V vs SCE for the functionalized monomers, as shown in Table 1. A cyclic voltammogram for **Indole-6-F_6_** is shown in Figure 1, where it is possible to see the maximum peak of oxidation of the monomer. Then, the polymerization was followed from −0.7 V to a potential slightly lower than *E*^ox^ (working potential *E*^w^) by the same electrochemical method. Examples of cyclic voltammograms for the polyindoles are presented in Figure 2.

**Table 1:** Oxidation potential (*E*^ox^) and working potential (*E*^w^) for each monomer by electrochemical process. Electropolymerization in 0.1 M of acetonitrile/Bu_4_NClO_4_.

monomer	oxidation potential *E*^ox^ (V)	working potential *E*^w^ (V)

**Indole**	1.64	1.56
**Indole-4-F_4_**	1.30	1.23
**Indole-5-F_4_**	1.31	1.26
**Indole-6-F_4_**	1.13	1.08
**Indole-4-F_6_**	1.19	1.13
**Indole-5-F_6_**	1.15	1.10
**Indole-6-F_6_**	1.07	1.03
**Indole-4-F_8_**	1.14	1.08
**Indole-5-F_8_**	1.06	1.01
**Indole-6-F_8_**	0.99	0.96

These cyclic voltammograms show only little variation in the polymer oxidation and reduction peaks due to the low conductivity of the polymers. Also, the short length of the new oligomers formed during the electropolymerization process increases their solubility resulting in polymeric films with a very low thickness. This may be explained by the reaction of amine groups of indole with the H^+^ ions released during the electropolymerization process. For these reasons, the deposition method has been changed and the depositions have been then performed at constant potential and using different normalized deposition charges, *Qs*, (from 0 to 100 mC·cm^−2^) in order to better control the amount of polymer electrodeposited. However, polymeric films were obtained with all the monomers except that with C_8_F_17_ chains because their large size induced very huge steric hindrances during the reaction and the polymerization is not favorable.

### Surface structures and wettability

**Figure 3:**
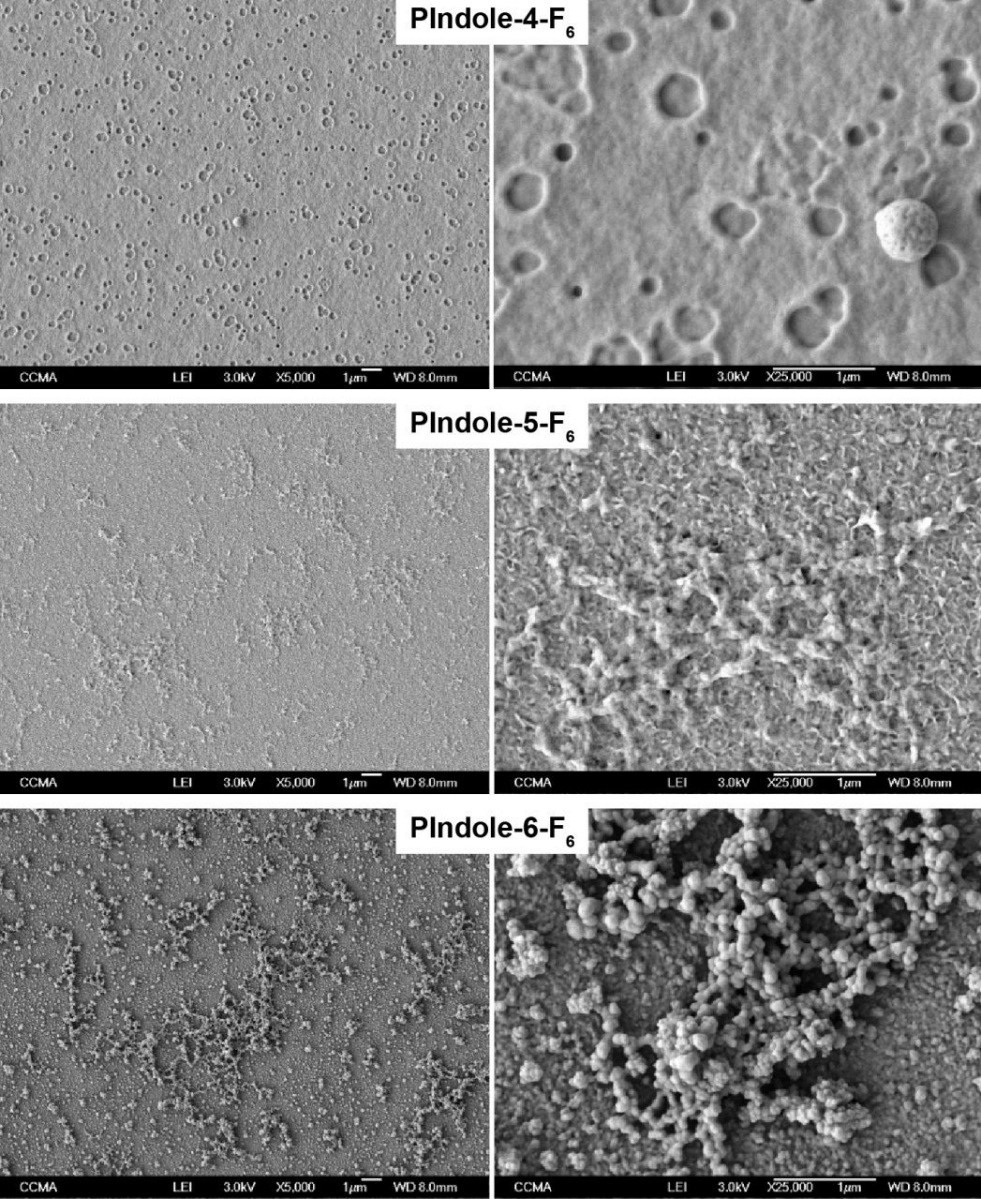
SEM images at two different magnifications (5000× and 25000×) of the polyindoles substituted with C_6_F_13_ chains in the different positions.

**Figure 4:**
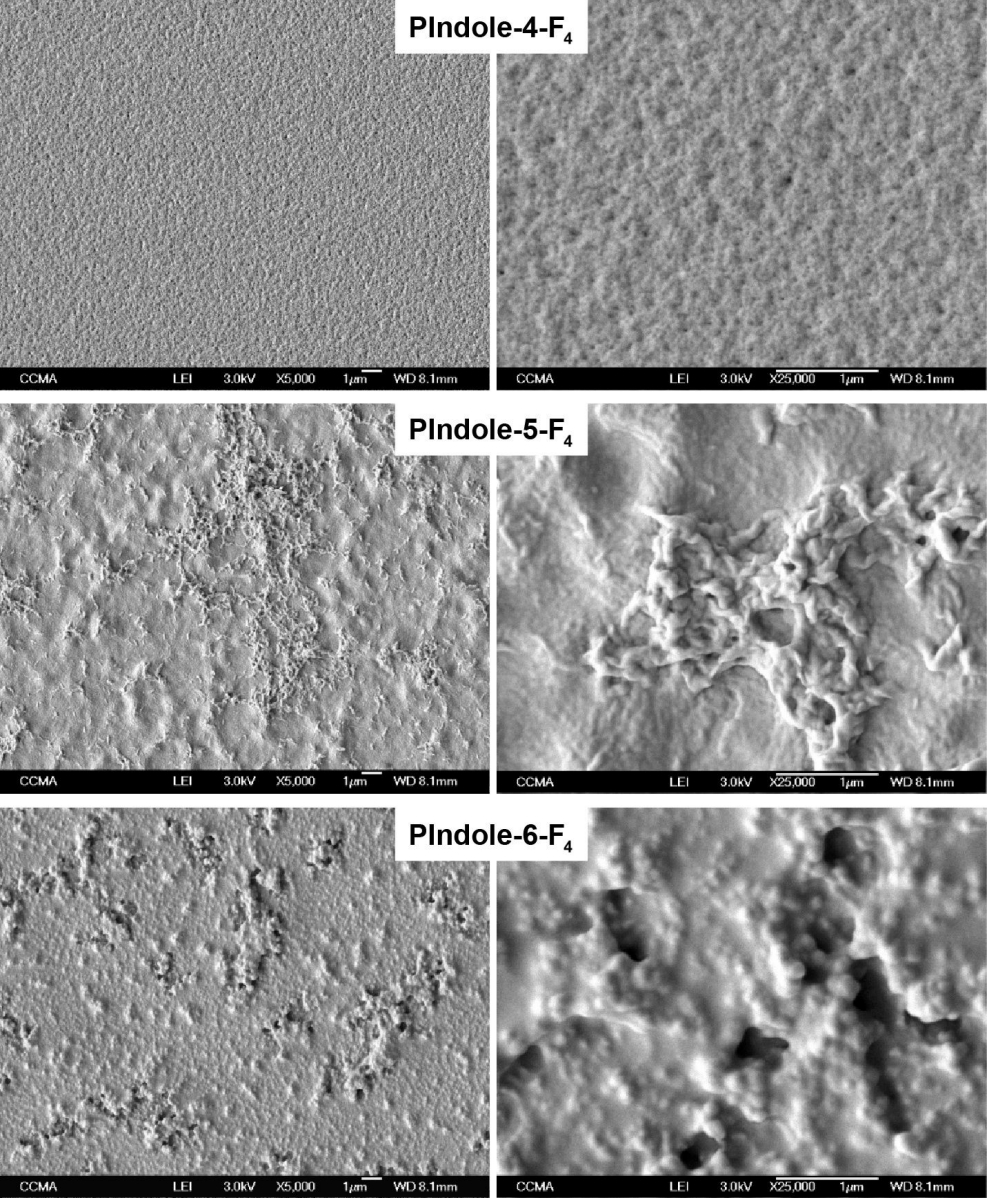
SEM images at two different magnifications (5000× and 25000×) of the polyindoles substituted with C_4_F_9_ chains in the different positions.

The surface structures were characterized by scanning electron microscopy (SEM) and surface roughness measurements. The SEM images for *Qs* = 100 mC·cm^−2^ are given in Figure 3 and Figure 4 and the surface roughness measurements can be found in Table 2. First of all, the surfaces are not very rough, however differences were observed especially with the substituent positions. Even if some craters are observed on **PIndole-4-F_6_**, the substitution in the 4-position gives the less structured surfaces. By contrast, nanofibers are observed with the substituents in the 5-position (**PIndole-5-F*_n_***) and spherical particles in the 6-position (**PIndole-6-F*_n_***). This confirms previous works in which authors showed that the polymerization is favorable in the indole positions 2, 3, 5 and 7 [31–32]. Indeed, if the polymerization of indole is more favorable in certain positions, the location of the substituent may influence the polymerization and the way in which the monomers are linked to one another forming different structures. This work is also in agreement with the literature where the authors showed that due to preferable polymerization positions on indole, fiber structures can be obtained by interfacial polymerization because the polymerization is directional, while spheres are obtained when the polymerization is equal in all directions [33]. In this case, the polymerization of the fluorinated indoles seems to be directional for **PIndole-5-F*_n_*** and proceeds equally in all directions for **PIndole-6-F*_n_***. For **PIndole-4-F*_n_***, the polymerization should not be favorable to form any structure on the surface. Previous works showed that one of the main parameters governing the surface roughness is the solubility of the oligomers formed in the first instance [26,34]. Hence, higher roughness of **PIndole-6-F*_n_*** can be explained by the formation of longer polymer chains. **PIndole-5-F_4_** and **PIndole-6-F_4_** also showed an increase in roughness for normalized charges of 50 and 100 mC·cm^−2^ without significant changes in the wettability comparing the others polyindoles. In contrast, **PIndole-4-F_6_** showed the same tendency as the other polymers in terms of conserving the same wettability even if their roughness only exhibited a significant increase at a normalized charge of 100 mC·cm^−2^.

**Table 2:** Arithmetic roughness (*R*_a_), quadratic roughness (*R*_q_) and apparent contact angles (θ) for the four probe liquids (water, diiodomethane, sunflower oil and hexadecane) for the polymers as a function of the normalized deposition charge.

polymer	normalized deposition charge (mC·cm^−2^)	*R*_a_ (nm)	*R*_q_ (nm)	θ_water_	θ_diiodomethane_	θ_sunflower_	θ_hexadecane_

**PIndole**	12.5	13.0	15.9	73.2	30.5	13.3	0
	25	10.8	13.2	71.5	25.3	12.0	0
	50	14.3	16.9	71.5	23.0	14.6	0
	100	21.9	31.0	69.8	27.2	10.9	0

**PIndole-4-F_6_**	12.5	15.5	19.5	109.2	83.1	57.7	65.1
	25	12.9	15.8	110.0	89.3	65.7	69.3
	50	12.7	15.2	112.4	88.8	77.0	72.4
	100	45.2	65.4	109.9	82.3	68.1	72.0

**PIndole-5-F_6_**	12.5	8.7	13.4	117.1	98.7	78.8	75.3
	25	9.5	19.0	116.3	100.7	92.5	73.8
	50	8.5	18.1	122.1	99.8	96.8	77.5
	100	17.7	31.2	124.8	103.3	87.0	74.8

**PIndole-6-F_6_**	12.5	17.5	25.3	146.5	106.5	92.1	79.8
	25	11.6	18.3	149.3	111.3	86.9	87.7
	50	22.1	33.4	159.0	116.3	107.1	93.3
	100	26.3	38.9	158.9	117.6	107.3	92.6

**PIndole-4-F_4_**	12.5	17.0	20.8	96.7	75.2	39.6	45.6
	25	16.5	20.2	99.5	83.3	59.4	52.3
	50	16.6	19.6	100.2	78.8	53.4	60.3
	100	15.4	18.9	98.7	78.1	59.6	43.4

**PIndole-5-F_4_**	12.5	36.8	50.2	107.6	85.7	68.0	48.8
	25	50.2	67.0	106.4	86.9	84.6	74.4
	50	117.0	158.9	108.4	85.7	70.2	61.4
	100	150.6	194.2	106.0	92.6	75.0	71.1

**PIndole-6-F_4_**	12.5	76.1	99.8	127.1	114.9	97.1	88.6
	25	71.5	98.8	142.9	122.7	106.7	97.7
	50	174.5	243.3	133.1	119.5	103.3	93.3
	100	153.3	202.9	131.2	120.2	100.8	93.4

**Figure 5:**
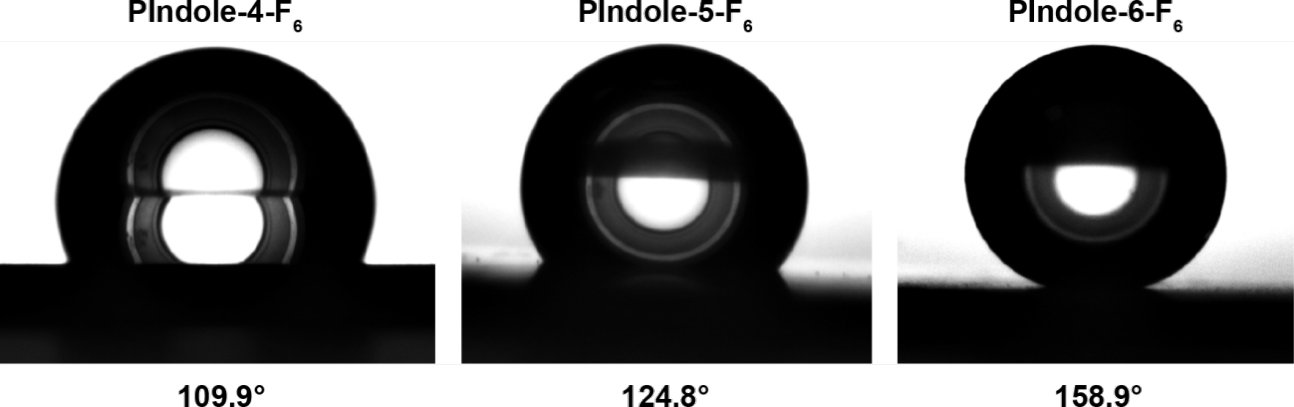
Image of a water droplet deposited on **PIndole-4-F_6_**, **PIndole-5-F_6_** and **PIndole-4-F_6_**; *Qs* = 100 mC·cm^−2^.

The wettability properties (Table 2) are in agreement with the SEM images. The polymers **PIndole-4-F*_n_*** are just slightly hydrophobic confirming the low effect of the surface structures for these polymers, independent of the fluorinated chain size. The polymers **PIndole-5-F*_n_*** are more hydrophobic with apparent water contact angles (θ_water_) of 124.8° for a normalized deposition charge of 100 mC·cm^−2^. Here, the contact angles are not very high because the nanofibers are horizontally aligned on the substrate. By contrast, the polymers **PIndole-6-F*_n_*** display extremely high θ_water_ and also superhydrophobic properties for **PIndole-6-F_6_**, even with a low roughness. The differences between the θ_water_ for the C_6_F_13_-polyindoles can be seen in Figure 5. Indeed, not only θ_water_ of 159.0° were measured on this polymer, but also highly oleophobic properties with θ_hexadecane_ = 93.3°. Moreover, the polymer presents extremely low hysteresis and sliding angles for normalized charges of 50 and 100 mC·cm^−2^, as shown in Table 3. For all polymers, the wettability changed with the position of the substituent on indole due to the differences in the structuration and with the increase of the length of the fluorinated chains. The different normalized charges did not result in large variations of wettability for any probe liquid, however, the highest normalized charge (100 mC·cm^−2^) presented the best results for wettability and roughness in almost all polyindoles.

**Table 3:** Dynamic water contact angles (hysteresis *H* and sliding angle α) for **PIndole-6-F_6_** as a function of the normalized deposition charge.

normalized deposition charge (mC·cm^−2^)	*H* (°)	α (°)

12.5	sticky behavior	
25	sticky behavior	
50	2.1	20.6
100	0.8	9.5

In order to explain the effects of the surface structures on the wetting properties, it is first necessary to prepare smooth substrates with each polymer and determine the contact angles (θ^Y^) for each probe liquid. These contact angles are dependent on the solid–vapor (γ_SV_), solid–liquid (γ_SL_) and liquid–vapor (γ_LV_) surface tensions following the Young equation [35] (cos θ^Y^ = (γ_SV_ − γ_SL_)/γ_LV_). The smooth substrates were obtained by reducing the normalized deposition charge (*Qs*) to 1 mC·cm^−2^ in order to cover all the substrate by a very thin polymer layer while avoiding the formation of surface structures. The roughness and the apparent contact angles of these smooth substrates are given in Table 4 confirming their ultra-low roughness and wettability. These results show that the polymers with C_6_F_13_ fluorinated chains are intrinsically hydrophobic (θ^Y^_water_ > 90°) while the polymers with C_4_F_9_ fluorinated chains as well as the polyindoles without fluorinated chain are slightly hydrophilic (θ^Y^_water_ < 90°). As expected the polymers with C_6_F_13_ fluorinated chains have also the highest oleophobicity even if the oil contact angles are relatively low.

**Table 4:** Arithmetic roughness (*R*_a_), quadratic roughness (*R*_q_) and apparent contact angles (θ) for the four probe liquids (water, diiodomethane, sunflower oil and hexadecane) for the “smooth” polymers.

polymer	*R*_a_ (nm)	*R*_q_ (nm)	θ^Y^_water_	θ^Y^_diiodomethane_	θ^Y^_sunflower_	θ^Y^_hexadecane_

**PIndole**	6.3	8.8	79.9	47.3	0	0
**PIndole-4-F_6_**	6.6	9.5	97.5	51.3	41.5	25.6
**PIndole-4-F_4_**	7.3	9.4	78.7	35.6	16.0	0
**PIndole-5-F_6_**	6.4	9.8	96.8	60.4	47.3	37.9
**PIndole-5-F_4_**	6.3	8.1	81.4	44.9	0	0
**PIndole-6-F_6_**	6.8	9.5	99.4	66.8	50.7	44.1
**PIndole-6-F_4_**	7.8	10.1	81.1	45.7	19.5	0

Indeed, two equations (the Wenzel and the Cassie–Baxter equation) [36–37] depending on θ^Y^ are very often used to explain the effect of the surface roughness on the wetting properties. In the Wenzel equation [36] (cos θ = *r*·cos θ^Y^, where *r* is a roughness parameter), the surface roughness can increase θ, but only if θ^Y^ > 90°. Hence, it is possible to have an extremely high θ_water_, but the contact angle hysteresis (*H*) is usually high because the surface roughness increases also the solid–liquid interface and thereby, increasing the adhesion between the water drop and the surface. Only the Cassie–Baxter equation [37] (cos θ = *r*_f_*·f*·cos θ^Y^ + *f* − 1, where *r*_f_ is the roughness ratio of the substrate wetted by the liquid, *f* the solid fraction and (1 − *f*) the air fraction) can predict the superhydrophobicity of **PIndole-6-F_6_**, for example. Here, the presence of a high amount of air between the droplet and the substrate can lead to extremely high θ_water_ with a very low *H*. In the case of **PIndole-6-F_6_**, the presence of the spherical nanoparticles formed on the surface during the polymerization allows to trap a high amount of air leading to superhydrophobic properties. These nanoparticles also induce a high increase of the surface oleophobicity, for example an increase of θ_hexadecane_ of 49.2°, from 44.1° on the smooth surface to 99.3° on the structured surface.

## Conclusion

Here we report for the first time the possibility to obtain hydrophobic and superhydrophobic polymeric films with a very low roughness by electropolymerization of fluorinated indoles differing by the length (C_4_F_9_, C_6_F_13_ and C_8_F_17_) and the position (4, 5 and 6-position on indole) of the perfluorinated substituent. Polymeric films were obtained for C_4_F_9_ and C_6_F_13_ showing several differences mainly with the substituent position, affecting the surface morphology and the wetting properties. The best results were obtained with **PIndole-6-F_6_** for which a superhydrophobic state with a self-cleaning condition and highly oleophobic properties were reached due to the presence of spherical nanoparticles and the fluorinated compounds on the surface. This work opens new ways in the formation of superhydrophobic polyindoles films by electrodeposition for future applications.

## Experimental

### Monomer synthesis and characterization

**Scheme 2:**
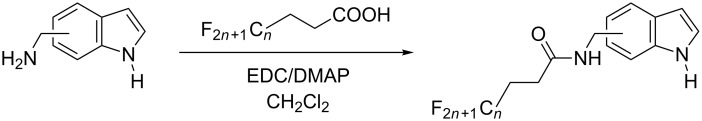
Synthesis way to the indole derivatives.

4-(aminomethyl)indole, 5-(aminomethyl)indole and 6-(aminomethyl)indole were purchased from Sigma-Aldrich. The monomers were synthesized by amidification between the corresponding (aminomethyl)indole and fluorinated acid (Scheme 2). More precisely, 0.26 g (1.4 mmol, 1 equiv) of *N*-(3-dimethylaminopropyl)-*N*′-ethylcarbodiimide hydrochloride (EDC) and 0.17 g of 4-dimethylaminopyridine (DMAP) (1.4 mmol, 1 equiv) were added to 20 mL of dichloromethane containing 1 equiv of the corresponding fluorinated acid. After stirring for 30 min, 0.2 g (1.4 mmol, 1 equiv) of the corresponding (aminomethyl)indole was added. The solution was stirred at room temperature for 24 h. The crude product was purified by column chromatography (stationary phase: silica gel; eluent: chloroform/methanol 95:5).

***N*-((1*H*-Indol-4-yl)methyl)-4,4,5,5,6,6,7,7,8,8,9,9,10,10,11,11,11-heptadecafluoroundecanamide (Indole-4-F_8_):** Yield 15%; yellow solid; mp 163.2 °C; ^1^H NMR (200 MHz, CD_3_OD) δ 7.32 (d, *J* = 8.0 Hz, 1H), 7.24 (d, *J* = 3.2 Hz, 1H), 7.06 (m, 1H), 6.93 (d, *J* = 6.8 Hz, 1H), 6.51 (dd, *J* = 3.2, 0.9 Hz, 1H), 4.65 (s, 2H), 2.55 (m, 4H); ^19^F NMR (188 MHz, CD_3_OD) δ −82.38 (m, 3F), −115.74 (m, 2F), −122.87 (m, 6F), −123.75 (m, 2F), −124.52 (m, 2F), −127.29 (m, 2F); ^13^C NMR (50 MHz, CD_3_OD) δ 172.45, 137.84, 130.34, 128.04, 125.64, 122.22, 119.39, 111.83, 100.41, 42.98, 28.1 (t, *J* = 23.1 Hz), 27.62 (t, *J* = 6.2 Hz); MS (70 eV) *m*/*z*: M^+^ 620 (82), C_9_H_9_N_2_^+•^ 145 (100), C_9_H_8_N^+•^ 130 (92), C_8_H_8_N^+^ 118 (52).

***N*-((1*H*-Indol-4-yl)methyl)-4,4,5,5,6,6,7,7,8,8,9,9,9-tridecafluorononanamide (Indole-4-F_6_):** Yield 25%; yellow solid; mp 150.2 °C; ^1^H NMR (200 MHz, CD_3_OD) δ 7.32 (d, *J* = 8.0 Hz, 1H), 7.24 (d, *J* = 3.2 Hz, 1H), 7.06 (m, 1H), 6.92 (d, *J* = 7.0 Hz, 1H), 6.50 (dd, *J* = 3.2, 0.9 Hz, 1H), 4.65 (s, 2H), 2.55 (m, 4H); ^19^F NMR (188 MHz, CD_3_OD) δ −82.43 (m, 3F), −115.74 (m, 2F), −122.93 (m, 2F), −123.91 (m, 2F), −124.60 (m, 2F), −127.36 (m, 2F); ^13^C NMR (50 MHz, CD_3_OD) δ 172.43, 137.81, 130.32, 128.04, 125.64, 122.22, 119.38, 111.82, 100.41, 42.98, 27.90 (t, *J* = 22.5 Hz), 27.47 (t, *J* = 2.8 Hz); MS (70 eV) *m*/*z*: M^+^ 520 (25), C_9_H_9_N_2_^+•^ 145 (100), C_9_H_8_N^+•^ 130 (87), C_8_H_8_N^+^ 118 (59).

***N*-((1*H*-Indol-4-yl)methyl)-4,4,5,5,6,6,7,7,8,8,8-undecafluorooctanamide (Indole-4-F_4_):** Yield 21%; yellow solid; mp 139.8 °C; ^1^H NMR (200 MHz, CD_3_OD) δ 7.32 (d, *J* = 8.1 Hz, 1H), 7.24 (d, *J* = 3.2 Hz, 1H), 7.06 (m, 1H), 6.93 (d, *J* = 7.6 Hz, 1H), 6.50 (dd, *J* = 3.2, 0.9 Hz, 1H), 4.65 (s, 2H), 2.55 (m, 4H); ^19^F NMR (188 MHz, CD_3_OD) δ −82.69 (m, 3F), −115.98 (m, 2F), −125.63 (m, 2F), −127.31 (m, 2F); ^13^C NMR (50 MHz, CD_3_OD) δ 172.37, 137.75, 130.26, 127.98, 125.58, 122.16, 119.32, 111.76, 100.35, 42.91, 27.82 (t, *J* = 22.1 Hz), 27.50 (t, *J* = 4.0 Hz); MS (70 eV) *m*/*z*: M^+^ 420 (40), C_9_H_9_N_2_^+•^ 145 (95), C_9_H_8_N^+•^ 130 (100), C_8_H_8_N^+^ 118 (65).

***N*-((1*H*-Indol-5-yl)methyl)-4,4,5,5,6,6,7,7,8,8,9,9,10,10,11,11,11-heptadecafluoroundecanamide (Indole-5-F_8_):** Yield 28%; yellow solid; mp 135.9 °C; ^1^H NMR (200 MHz, CD_3_OD) δ 7.47 (s, 1H), 7.33 (d, *J* = 8.4 Hz, 1H), 7.21 (d, *J* = 3.1 Hz, 1H), 7.04 (dd, *J* = 8.4, 1.6 Hz, 1H), 6.40 (dd, *J* = 3.1, 0.8 Hz, 1H), 4.49 (s, 2H), 2.53 (m, 4H); ^19^F NMR (188 MHz, CD_3_OD) δ −82.40 (m, 3F), −115.74 (m, 2F), −122.90 (m, 6F), −123.77 (m, 2F), −124.60 (m, 2F), −127.36 (m, 2F); ^13^C NMR (50 MHz, CD_3_OD) δ 172.32, 137.03, 129.90, 129.53, 126.06, 122.45, 120.51, 112.24, 102.24, 45.09, 27.82 (t, *J* = 21.4 Hz), 27.53 (t, *J* = 4.1 Hz); MS (70 eV) *m*/*z*: M^+^ 620 (4), C_9_H_9_N_2_^+•^ 145 (100), C_9_H_8_N^+•^ 130 (92), C_8_H_8_N^+^ 118 (47).

***N*-((1*H*-Indol-5-yl)methyl)-4,4,5,5,6,6,7,7,8,8,9,9,9-tridecafluorononanamide (Indole-5-F_6_):** Yield 51%; yellow solid; mp 85.5 °C; ^1^H NMR (200 MHz, CD_3_OD) δ 7.47 (s, 1H), 7.33 (d, *J* = 8.3 Hz, 1H), 7.21 (d, *J* = 3.1 Hz, 1H), 7.03 (dd, *J* = 8.3, 1.6 Hz, 1H), 6.40 (dd, *J* = 3.1 Hz, 0.8 Hz, 1H), 4.44 (s, 2H), 2.47 (m, 4H); ^19^F NMR (188 MHz, CD_3_OD) δ −82.45 (m, 3F), −115.74 (m, 2F), −122.95 (m, 2F), −123.93 (m, 2F), −124.60 (m, 2F), −127.36 (m, 2F); ^13^C NMR (50 MHz, CD_3_OD) δ 172.38, 137.10, 129.97, 129.61, 126.13, 122.52, 120.58, 112.31, 102.31, 45.16, 27.86 (t, *J* = 22.8 Hz), 27.60 (t, *J* = 3.0 Hz); MS (70 eV) *m*/*z*: M^+^ 520 (35), C_9_H_9_N_2_^+•^ 145 (100), C_9_H_8_N^+•^ 130 (90), C_8_H_8_N^+^ 118 (54).

***N*-((1*H*-Indol-5-yl)methyl)-4,4,5,5,6,6,7,7,8,8,8-undecafluorooctanamide (Indole-5-F_4_):** Yield 61%; yellow solid; mp 47.7 °C; ^1^H NMR (200 MHz, CD_3_OD) δ 7.47 (s, 1H), 7.33 (d, *J* = 8.4 Hz, 1H), 7.21 (d, *J* = 3.1 Hz, 1H), 7.04 (dd, *J* = 8.4, 1.6 Hz, 1H), 6.40 (dd, *J* = 3.1, 0.8 Hz, 1H), 4.44 (s, 2H), 2.47 (m, 4H); ^19^F NMR (188 MHz, CD_3_OD) δ −82.67 (m, 3F), −115.97 (m, 2F), −125.60 (m, 2F), −127.21 (m, 2F); ^13^C NMR (50 MHz, CD_3_OD) δ 172.32, 137.03, 129.91, 129.54, 126.07, 122.45, 120.52, 112.24, 102.25, 45.09, 27.78 (t, *J* = 22.5 Hz), 27.64 (t, *J* = 3.9 Hz); MS (70 eV) *m*/*z*: M^+^ 420 (98), C_9_H_9_N_2_^+•^ 145 (90), C_9_H_8_N^+•^ 130 (100), C_8_H_8_N^+^ 118 (70).

***N*-((1*H*-Indol-6-yl)methyl)-4,4,5,5,6,6,7,7,8,8,9,9,10,10,11,11,11-heptadecafluoroundecanamide (Indole-6-F_8_):** Yield 30%; yellow solid; mp 121.0 °C; ^1^H NMR (200 MHz, CD_3_OD) δ 7.49 (d, *J* = 8.1 Hz, 1H), 7.32 (s, 1H), 7.20 (d, *J* = 3.1 Hz, 1H), 6.95 (dd, *J* = 8.1, 1.4 Hz, 1H), 6.40 (dd, *J* = 3.1, 0.8 Hz, 1H), 4.45 (s, 2H), 2.56 (m, 4H); ^19^F NMR (188 MHz, CD_3_OD) δ −82.39 (m, 3F), −115.76 (m, 2F), −122.86 (m, 6F), −123.78 (m, 2F), −124.58 (m, 2F), −127.29 (m, 2F); ^13^C NMR (50 MHz, CD_3_OD) δ 172.36, 137.73, 132.46, 128.79, 125.87, 121.30, 120.22, 111.47, 102.17, 45.10, 27.79 (t, *J* = 21.0 Hz), 27.43 (t, *J* = 5.0 Hz); MS (70 eV) *m*/*z*: M^+^ 620 (4), C_9_H_9_N_2_^+•^ 145 (100), C_9_H_8_N^+•^ 130 (97), C_8_H_8_N^+^ 118 (45).

***N*-((1*H*-Indol-6-yl)methyl)-4,4,5,5,6,6,7,7,8,8,9,9,9-tridecafluorononanamide (Indole-6-F_6_):** Yield 19%; yellow solid; mp 120.7 °C; ^1^H NMR (200 MHz, CD_3_OD) δ 7.49 (d, *J* = 8.1 Hz, 1H), 7.31 (s, 1H), 7.20 (d, *J* = 3.1 Hz, 1H), 6.94 (dd, *J* = 8.1, 1.5 Hz, 1H), 6.40 (dd, *J* = 3.1, 0.8 Hz, 1H), 4.45 (s, 2H), 2.47 (m, 4H); ^19^F NMR (188 MHz, CD_3_OD) δ −82.45 (m, 3F), −115.77 (m, 2F), −122.95 (m, 2F), −123.93 (m, 2F), −124.60 (m, 2F), −127.36 (m, 2F); ^13^C NMR (50 MHz, CD_3_OD) δ 172.43, 137.80, 132.52, 128.86, 125.93, 121.36, 120.28, 111.54, 102.23, 45.15, 27.88 (t, *J* = 21.0 Hz), 27.58 (t, *J* = 3.5 Hz); MS (70 eV) *m*/*z*: M^+^ 520 (40), C_9_H_9_N_2_^+•^ 145 (100), C_9_H_8_N^+•^ 130 (94), C_8_H_8_N^+^ 118 (53).

***N*-((1*H*-Indol-6-yl)methyl)-4,4,5,5,6,6,7,7,8,8,8-undecafluorooctanamide (Indole-6-F_4_):** Yield 33%; yellow solid; mp 105.7 °C; ^1^H NMR (200 MHz, CD_3_OD) δ 7.50 (d, *J* = 8.1 Hz, 1H), 7.31 (s, 1H), 7.20 (d, *J* = 3.2 Hz, 1H), 6.95 (dd, *J* = 8.1, 1.5 Hz, 1H), 6.40 (dd, *J* = 3.1, 0.8 Hz, 1H), 4.45 (s, 2H), 2.58 (m, 4H); ^19^F NMR (188 MHz, CD_3_OD) δ −82.66 (m, 3F), −115.91 (m, 2F), −125.60 (m, 2H), −127.27 (m, 2H); ^13^C NMR (50 MHz, CD_3_OD) δ 172.43, 137.72, 132.45, 128.78, 125.87, 121.29, 120.21, 111.47, 102.21, 45.08, 27.72 (t, *J* = 21.0 Hz), 27.56 (t, *J* = 4.0 Hz); MS (70 eV) *m*/*z*: M^+^ 420 (85), C_9_H_9_N_2_^+•^ 145 (85), C_9_H_8_N^+•^ 130 (100), C_8_H_8_N^+^ 118 (70).

### Electrodeposition parameters

The polyindole films were electrodeposited by using a potentiostat (Autolab). For this, 2 cm^2^ gold plates were chosen as working electrode, a carbon rod as counter-electrode while saturated calomel (SCE) was taken as reference electrode. The electrolyte used was a 0.1 mol solution of tetrabutylammonium perchlorate (Bu_4_NClO_4_) in anhydrous acetonitrile. Before the electrodeposition, the solution was degassed under argon and 0.01 mol of monomer was introduced. After the electrodeposition, the coated substrates were washed three times in acetonitrile and slowly dried.

### Polymer and surface characterization

The surface roughness (arithmetic *R*_a_ and quadratic *R*_q_) were determined by using a Wyko NT 1100 optical microscope of Bruker. The data were obtained using the High Mag Phase Shift Interference (PSI) working mode, the objective 50× and the field of view (FOV) 0.5×.

The scanning electron microscopy images were obtained by using a 6700F microscope of JEOL.

The contact angles were determined by using a DSA30 goniometer of Krüss. Liquids of different surface tension were chosen to characterize the surface hydrophobicity and oleophobicity: water (γ_LV_ = 72.8 mN·m^−1^), diiodomethane (γ_LV_ = 50.0 mN·m^−1^), sunflower oil (γ_LV_ ≈ 31 mN·m^−1^) and hexadecane (γ_LV_ = 27.6 mN·m^−1^). The apparent contact angles (θ) were obtained by taken the angle at the triple point of a liquid droplet put on the substrate. The contact angle hysteresis (*H*) and sliding angle (α) were determined with the tilted-drop method. Here, a 6 µL liquid droplet was put on the substrate and the substrate was inclined until the droplet moving. The maximum inclination angle is α. The advanced (θ_adv_) and receding (θ_rec_) contact angles and by deduction the hysteresis *H* = θ_adv_ – θ_rec_ were taken just before the moving of the droplet, the angle in the moving direction being θ_adv_ and that in the opposite direction θ_rec_.
